# Supplementary material for: Intrinsic properties of spinal motoneurons degrade ankle torque control in humans
Source: J Physiol. 2025 Mar 28;603(8):2443–63. doi: 10.1113/JP287446 (PMC12013794; doi:10.1113/JP287446)
Supplement: Supplementary file 2 — Supplementary Information [file TJP-603-2443-s001.docx]

**Supplementary Materials**

**
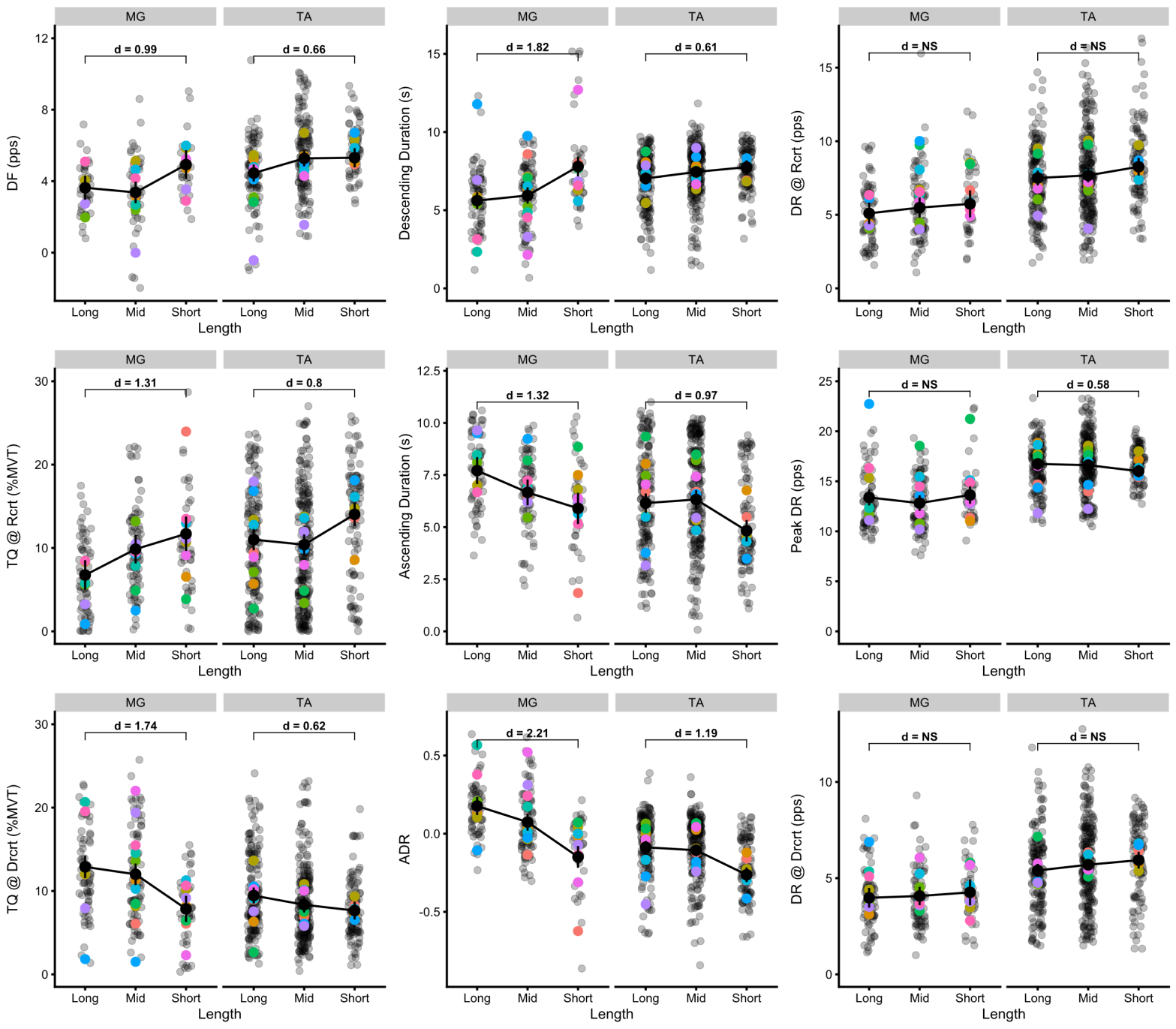
Supplementary Figures**

**Figure S1: Discharge characteristics for motor units matched between lengths.** The top row from left to right represent ΔF, the duration of time spent on the descending portion of the ramp, and discharge rate (DR) at motor unit recruitment (Rcrt). The middle row represents the torque (TQ) at motor unit recruitment, the time spent on the ascending portion of the ramp, and the peak discharge rate. The bottom row represents torque at derecruitment (Drcrt), the difference between ascending and descending duration as a function of the entire duration of discharge (ADR), and the discharge rate at derecruitment. Colored dots represent participant averages, grey dots represent raw data points for individual motor units, and the black connecting lines indicate the estimated marginal means predicted by the linear mixed effects model. Vertical black lines indicate the 95% confidence interval for these estimated marginal means. Cohen’s d effect size is shown for the difference between long and short when the differences are predicted as significant by the mixed model (p<0.05) and are quantified with the estimated marginal means. Significance for the fixed factor of length and its interaction with muscle is as follows: ΔF: (χ^2^(4) = 33.24, p < 0.001); Descending Duration: (χ^2^(4) = 69.58, p < 0.001); DR @ Rcrt: (χ^2^(4) = 7.74, p = 0.101); TQ @ Rcrt: (χ^2^(4) = 77.07, p < 0.001); Ascending Duration: (χ^2^(4) = 90.33, p < 0.001); Peak DR: (χ^2^(4) = 26.60, p < 0.001); TQ @ Drcrt: (χ^2^(4) = 64.94, p < 0.001); ADR: (χ^2^(4) = 136.06, p < 0.001); DR @ Drcrt: (χ^2^(4) = 5.04, p = 0.284).

**
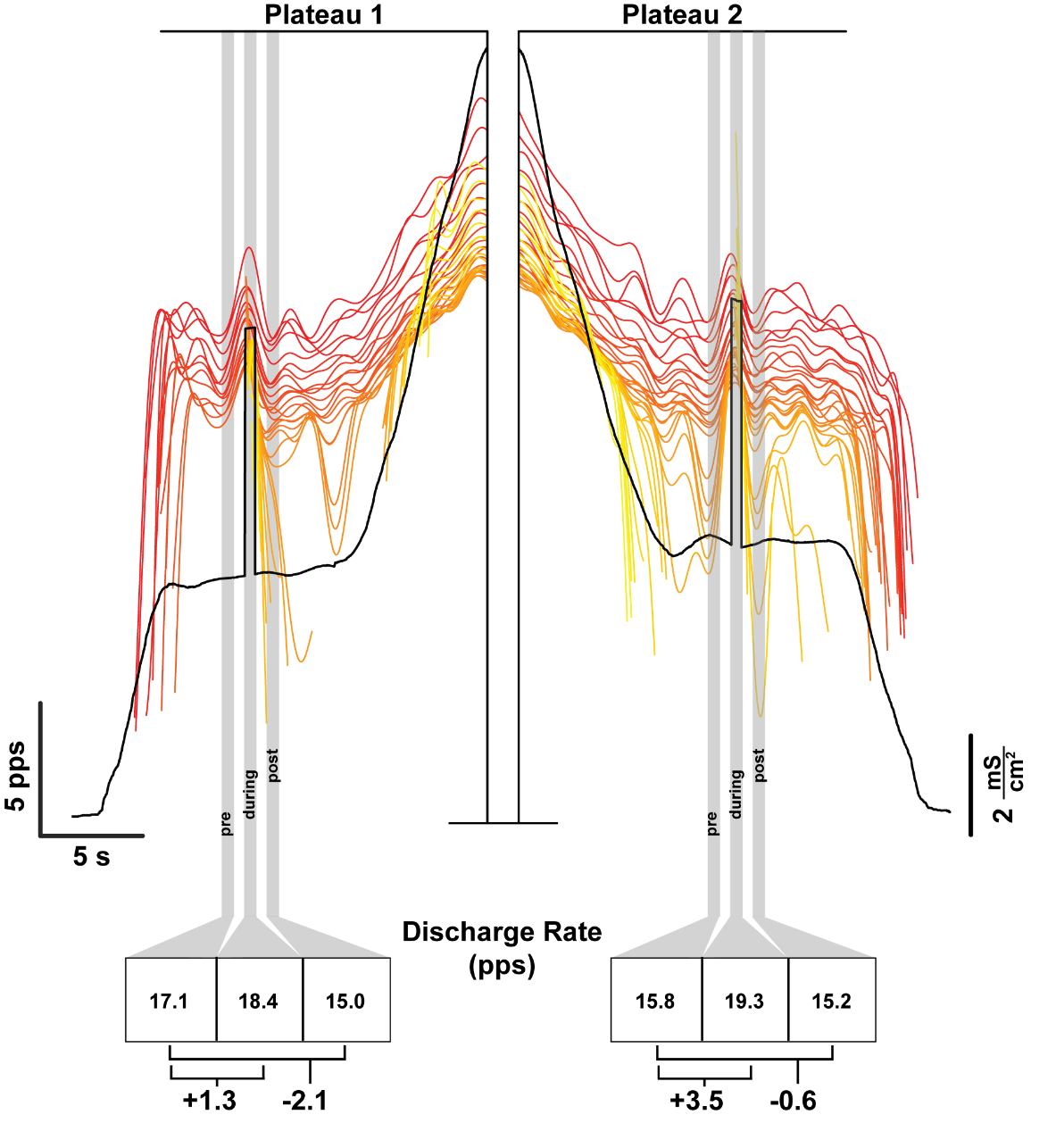
**

**Figure S2: Motoneuron output gain is greatest on the second plateau.** Smoothed discharge rates of twenty simulated motoneurons are shown in response to an excitatory input conductance (black trace). The input conductance is equivalent to the cumulative spike train of the tibialis anterior motor units at the mid-length sombrero. The left and right indicate trials where a 500 ms excitatory perturbation (conductance x 5) was applied to either the first or second plateau, respectively. The smoothed discharge rates in pulse-per-second (pps) are colored from low to high threshold (red-yellow). The average discharge rate across all motoneurons in 500 ms windows before, during, and after are shown across the bottom. As indicated, the change in discharge rate from before to during the excitatory perturbation is greatest during plateau two, indicating greater output gain.

**Supplementary Methods**

Supplementary Experiment 1:

Simulated motoneuron spike trains were generated with a pool of motoneurons modeled according to previously detailed methods. (Powers & Heckman, 2017a; Beauchamp *et al.*, 2023b; Chardon *et al.*, 2023b) The motoneuron pool consists of 20 model motoneurons to reflect the typical sample size of MUs discriminated with high-density surface EMG recordings, with each motoneuron possessing unique intrinsic properties. Each motoneuron was comprised of a soma and four dendritic compartments and possessed size and biophysical characteristics described previously.(Kim *et al.*, 2009; Beauchamp *et al.*, 2023b; Chardon *et al.*, 2023b) Briefly, sodium and potassium conductances were inserted into the soma, a calcium conductance mediating the slowly-activating PIC was inserted into each dendrite, and a hyperpolarization-activated mixed-cation conductance was inserted into all compartments. Conductance densities, kinetics, and steady-state activation curves were originally tuned to recreate the range of input-output behavior recorded in medial gastrocnemius motoneurons in decerebrate cats(Powers & Heckman, 2017a), and further augmented to represent the discharge behavior observed in the human.(Beauchamp *et al.*, 2023b; Chardon *et al.*, 2023b) (Powers & Heckman, 2015; Powers & Heckman, 2017b)

To create the simulations indicated in Figure S2, all motoneurons in the pool were driven with an excitatory input conductance equivalent to the cumulative spike train of all motor units decomposed in the tibialis anterior during the mid-length sombrero contraction. In two separate simulation trials, this excitatory input conductance was perturbed for 500 ms on either the first or the second plateau by multiplying the original conductance by five. During both simulations, the pattern of inhibition was adjusted as a function of excitation through dendritic inhibitory conductance in a reciprocal pattern (excitation times -0.5). The resultant motoneuron spike times were transformed into smooth discharge rate estimates with support vector regression as detailed previously. (Beauchamp *et al.*, 2022b)

All simulations were run using NEURON. (Hines & Carnevale, 1997) NEURON files specifying motoneuron pool parameters, conductance mechanisms, and protocols for producing motoneuron pool output in response to synaptic conductance inputs can be found at http://modeldb.yale.edu/239582.
